# Why childhood-onset type 1 diabetes impacts labour market outcomes: a mediation analysis

**DOI:** 10.1007/s00125-017-4472-3

**Published:** 2017-11-23

**Authors:** Sofie Persson, Gisela Dahlquist, Ulf-G. Gerdtham, Katarina Steen Carlsson, for the Swedish Childhood Diabetes Study Group

**Affiliations:** 1 Health Economics Unit, Department of Clinical Sciences, Malmö, Lund University, Medicon Village, 223 81 Lund, Sweden; 2 Department of Clinical Sciences, Paediatrics, Umeå University, Umeå, Sweden; 3 Department of Economics, Lund University, Lund, Sweden; 4 Centre for Economic Demography, Lund University, Lund, Sweden

**Keywords:** Children, Education, Inpatient care, Mediation analysis, Occupation, Outpatient care, Sickness benefits, Type 1 diabetes

## Abstract

**Aims/hypothesis:**

Previous studies show a negative effect of type 1 diabetes on labour market outcomes such as employment and earnings later in life. However, little is known about the mechanisms underlying these effects. This study aims to analyse the mediating role of adult health, education, occupation and family formation.

**Methods:**

A total of 4179 individuals from the Swedish Childhood Diabetes Register and 16,983 individuals forming a population control group born between 1962 and 1979 were followed between 30 and 50 years of age. The total effect of having type 1 diabetes was broken down into a direct effect and an indirect (mediating) effect using statistical mediation analysis. We also analysed whether type 1 diabetes has different effects on labour market outcome between the sexes and across socioeconomic status.

**Results:**

Childhood-onset type 1 diabetes had a negative impact on employment (OR 0.68 [95% CI 0.62, 0.76] and OR 0.76 [95% CI 0.67, 0.86]) and earnings (−6%, *p* < 0.001 and −8%, *p* < 0.001) for women and men, respectively. Each of the mediators studied contributed to the total effect with adult health and occupational field accounting for the largest part. However, some of the effect could not be attributed to any of the mediators studied and was therefore likely related to other characteristics of the disease that hamper career opportunities. The effect of type 1 diabetes on employment and earnings did not vary significantly according to socioeconomic status of the family (parental education and earnings).

**Conclusions/interpretation:**

A large part of the effect of type 1 diabetes on the labour market is attributed to adult health but there are other important mediating factors that need to be considered to reduce this negative effect.

**Electronic supplementary material:**

The online version of this article (10.1007/s00125-017-4472-3) contains peer-reviewed but unedited supplementary material, which is available to authorised users.

## Introduction

Previous studies report that living with type 1 diabetes can have a negative impact on labour market outcomes [1–4]. Childhood-onset type 1 diabetes has been estimated to reduce earnings by 9% and 10% for individuals aged 27–32 years [2] and onset of type 1 diabetes in adolescence has been estimated to reduce earnings by 8% and 4% for women and men, respectively, at 10 years after diagnosis [1]. The effect on earnings increases with age [5] and disease duration [2]. However, the mechanisms linking type 1 diabetes and labour market outcomes is poorly understood so further research is needed to help reduce the adverse impact of the disease in the future.

Several mechanisms through which type 1 diabetes may impact labour market outcomes have been suggested. First, the disease has a documented impact on several educational outcomes, such as school grades, total number of years of schooling and the likelihood of university education [2, 6–8]. Second, previous findings indicate that the choice of occupation and career opportunities may be impacted by the disease [2]. Third, reduced fertility and increased risk of pregnancy complications caused by type 1 diabetes [9, 10] may play a role in explaining the effect on labour market outcomes. Fourth, type 1 diabetes is a life-long chronic disease associated with the development of short-term complications such as hyperglycaemia with ketoacidosis and frequent hypoglycaemic episodes, together with micro- and macrovascular complications that develop over time [11]. The labour market effects may therefore operate through increased absenteeism, reduced work capacity and early retirement. Type 1 diabetes has previously been associated with more sick leave per year and decreased health-related quality of life [3] with several studies indeed reporting reduced work productivity and increased work absence due to hypoglycaemia [12–15].

The purpose of this study was to estimate the overall impact of childhood-onset type 1 diabetes on employment and earnings between 30 and 50 years of age and to break this effect down to explore the relative importance of four potential mediating factors: education, occupation, family formation and health. Moreover, we studied whether type 1 diabetes differentially impacts labour market outcomes across socioeconomic status groups.

## Methods

### Study population

The present study uses data from the Swedish Childhood Diabetes Register (SCDR), a research register in which incident Swedish cases of type 1 diabetes younger than 15 years [16] are prospectively registered to study risk factors for type 1 diabetes and its complications. Parents and/or children gave informed consent to the registration. The SCDR has been active since 1 July 1977 and has a high level of coverage (96–99%) [17, 18].

To study the long-term consequences of type 1 diabetes, the SCDR has been linked to several official administrative databases including health registers at the National Board of Health and Welfare and the following socioeconomic databases at Statistics Sweden; the Longitudinal Integration Database for Health Insurance and Labour Market Studies (LISA) [19]; the Swedish Register of Education [20] and the National Patient Register for in- and outpatient care [21]. Additionally, information was collected from the LISA database regarding the parents, who were identified through the Multi-Generation Register [22]. Linkage was performed using the Swedish personal identification number. Only coded data were made available to the researchers and the code key was kept at Statistics Sweden.

A control group for comparison was included using a matched case–control design whereby four individuals from the Swedish general population were matched to each person in the SCDR. Statistics Sweden performed the matching of these individuals based on year of birth and municipality of residence at the time of the corresponding individuals being diagnosed with type 1 diabetes.

The study was approved by the Regional Research Ethics Board at Umeå University (dnr 07-169 M), the National Board of Health and Statistics Sweden.

For this study, individuals born between 1962 (the earliest age available) and 1979 were selected. In total 4281 individuals with type 1 diabetes and 17,120 individuals forming the control group, were followed from 30 years of age (an age by which most people have reached their final educational level) until 50 years of age.

### Analysis

**Fig. 1:**
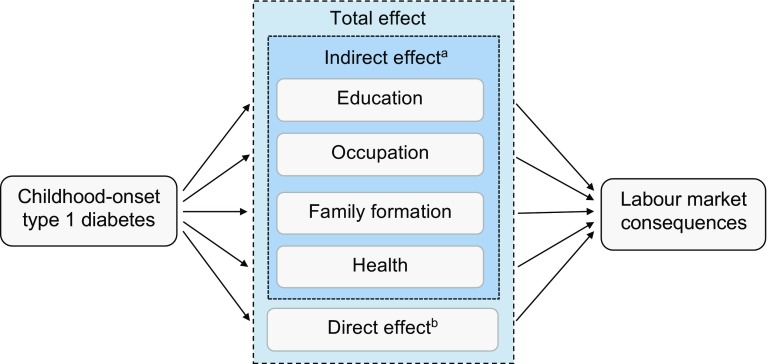
Conceptual framework of the mediation analysis**.** ^a^Measured possible mediators**;** ^b^represents all other possible explanations for the relationship between type 1 diabetes and labour market outcomes that were not capured by the studied mediators

Mediation analysis is a statistical method for identifying and explaining the possible mechanisms behind an observed relationship between two variables through a third variable (i.e. a mediator). Figure 1 outlines the mediation analysis framework in this study. It was hypothesised that part of the total effect of diabetes on labour market outcomes may operate through four mediating factors: education, occupation, family formation and health; referred to as the indirect effects. The remaining part of the total effect, not explained by these mediators, represents all other possible explanations for the relationship between diabetes and labour market outcomes; referred to as the direct effect [23].

The first step of the analysis explored the effect of diabetes on each of the potential mediators separately at 30 and 40 years of age. The second step used mediation analysis to estimate the total mean effect of diabetes on employment and earnings between 30 and 50 years of age and to break this down into an indirect effect (through the mediators) and a direct effect (not through the mediators). Demographic and socioeconomic background characteristics were controlled for in these analyses. The third step investigated whether the effect of diabetes differs across individuals with different parental socioeconomic status, by estimating interaction effects between diabetes and parents’ educational level and earnings.

### Variables

The two main outcome variables were employment (defined as employed or self-employed in November each year) and earnings if employed (annual earnings in Euro and deflated into 2013 prices, where EUR 1 = SEK 8.649 [24]). The presence of diabetes was defined either as a binary variable (0 = control group and 1 = diabetes case) or as a categorical variable for the duration of the disease (0 = control group, 1 ≤ 24 years and 2 ≥ 25 years).

Education was defined as total number of years of schooling and was calculated based on the highest educational level using the algorithm described by Gerdtham et al [25]. Occupational status was defined using the mean income in 2013 of each respective three-digit occupational category [26] according to the Swedish standard classification of occupations (the SSYK3 code). This information was available from 2001 and the occupational category for the closest available age was used for individuals born in 1970 or earlier (first value carried backwards). Family formation was defined as a binary variable for having at least one child in the household.

For the health mediator, three indicators were constructed based on sickness benefit data (accessible from the national social insurance system for ≥14 days of sick leave) from the LISA dataset and in- and outpatient hospital care data from the National Patient Register; (1) having received sickness benefits in the year; (2) having used inpatient care in the year and (3) having made two or more specialist outpatient care visits in the year. In Sweden, adults with type 1 diabetes commonly have one routine visit to their physician per year so the definition of two or more visits was used to capture an increased need for healthcare. The analysis of data reflecting outpatient care was restricted to the period 2004–2013, for those between 30 and 39 years of age, for reasons of data availability.

Variables regarding demographic and socioeconomic background were identified using information about parents’ country of birth and level of education and earnings. Parents’ level of educational was defined by the highest attained education (low = compulsory schooling; medium = upper secondary school; high = university; and ‘missing’). The earnings of the parents were defined as mean annual earnings during 1990–2013, deflated into 2013 prices [24]. Calendar year was controlled for by use of yearly dummy variables.

To analyse how the effects of type 1 diabetes differed according to socioeconomic status, education was defined as low if neither parent had completed upper secondary school education. Earnings were defined as low if both parents had lower than the median earnings of the parents in the study (<EUR 17,000 per year for mothers and <EUR 22,000 for fathers).

### Statistical analysis

Descriptive data were expressed as means (± SD) or median (min–max). The effect of diabetes on the mediators at 30 years of age was analysed using either ordinary least square (OLS) and logistic regression with two model specifications. Model 1 assessed the total effect of diabetes and Model 2 split diabetes into duration ≤24 years and ≥25 years, adjusting for demographic and socioeconomic background and calendar year. At 40 years of age, the duration was 25–35 years, with few observations made after 30 years of duration. Therefore, only the total effect of diabetes was estimated (Model 1) for this age group.

The Sobel–Goodman mediation test was used to investigate whether the mediators did in fact influence the effect of diabetes on employment and earnings. Thereafter, we followed the same approach as Tubeuf et al [27] and Damman et al [28] and utilised a method developed by Karlson, Holme and Breen (KHB method) [29–31] to investigate to what extent the relationship between diabetes and employment and earnings is mediated by each mediator. This method enabled us to break down the total effect of diabetes and to simultaneously investigate the respective contribution of each of the mediators. This is necessary when assessing mediators that are not independent of each other, which is likely to be the case here, to avoid replicating the contributions of each mediator [32]. The KHB method also adjusts for rescaling issues that may arise in cross-model comparison of non-linear models [29–31].

The mediation analysis was performed in a panel data setting with annual data from 30 years until the age of 50 years of age using logit and OLS regression with clustered standard errors, controlling for demographic and socioeconomic background and calendar year. The 95% CIs for the estimate effects were calculated using bootstrapping with 500 replicates [27, 33].

Sensitivity analyses were performed in accordance with recommendations by Imai et al [34–36] to test sensitivity to violations of the assumptions of causal mediation. See the electronic supplementary material (ESM) Methods, ESM Table 1 and ESM Figs 1–2 for further description and presentation of results.

All analyses were performed separately according to sex, in line with the labour economic and epidemiological literature. Analyses were performed using Stata version 14 (StataCorp, College Station, TX, USA).

## Results

### Descriptive statistics

**Fig. 2:**
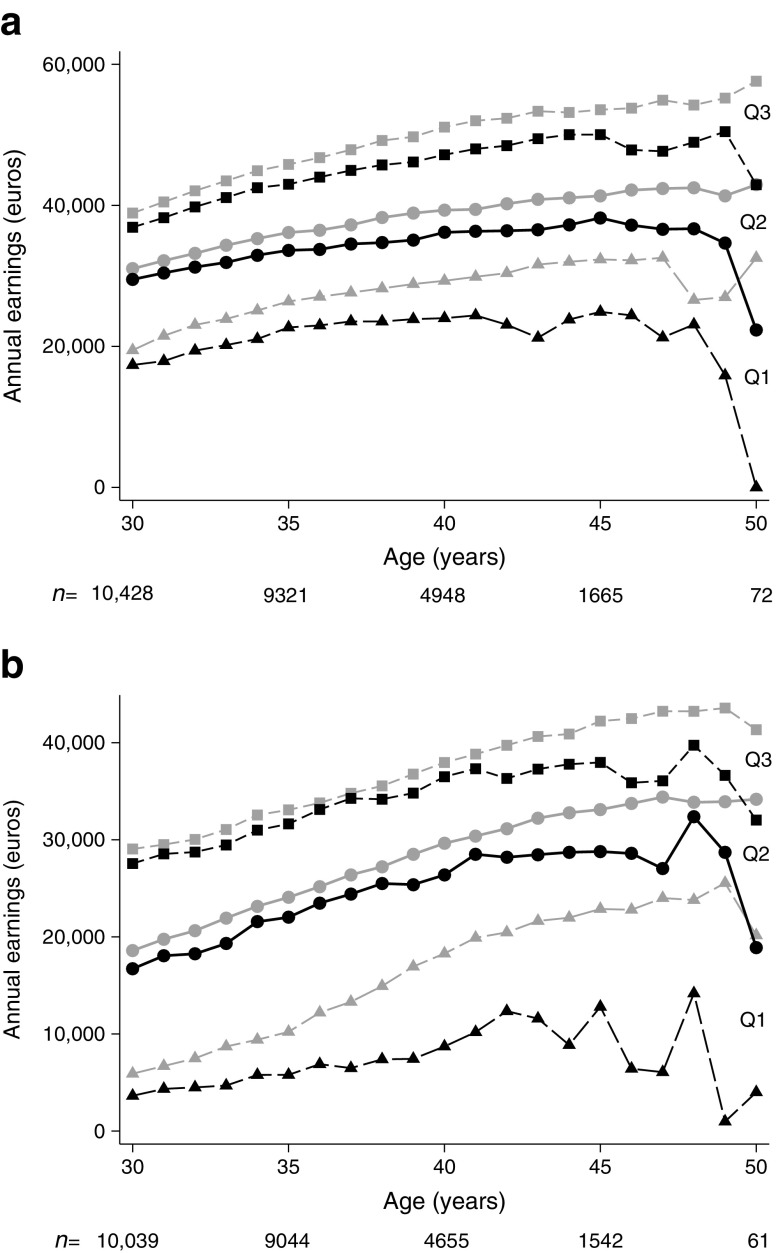
Annual earnings of men (**a**) and women (**b**) with type 1 diabetes (black) and the control group (grey) using first (Q1), second (median) (Q2), and third (Q3) quartiles from 30 to 50 years of age, deflated into 2013 prices. The number of individuals at each age is shown below the graph

Individuals with type 1 diabetes were diagnosed in 1977–1994 at the mean (±SD) age of 10.3 (±3.4) years (Table 1). At 30 years of age, a total of 4179 (97.6%) individuals with diabetes and 16,983 (99.2%) individuals in the control group were alive and included in the analysis (Table 2). The socioeconomic characteristics did not differ between the groups, except for the category ‘missing data’ for parental education, where the control group accounted for a larger proportion (*p* < 0.001). Additionally, the proportion of individuals within the control group with a parent born in a non-Nordic country was larger (*p* < 0.001). Unadjusted data showed lower earnings at 30 years of age and a seemingly increasing gap in earnings between the ages of 30 and 50 years (Fig. 2).

**Table 1:** Study population

	Type 1 diabetes group	Control group
Individuals	4179	16,983
Male sex, *n* (%)	2217 (53)	8509 (50)
Birth year, mean (min–max)	1973 (1962–1979)	1973 (1962–1979)
Year of diagnosis, mean (min–max)	1984 (1977–1994)	–
Age at diagnosis, mean (min–max)	10.3 (0–14.9)	–

**Table 2:** Characteristics of the study population at 30 years of age

	Women			Men		
Characteristic	Type 1 diabetes group	Control group	*p* ^a^	Type 1 diabetes group	Control group	*p* ^a^
Cohort born in 1962–1979, *n*	1962	8474		2217	8509	
Duration of diabetes, years, mean (min–max)	19.9 (15–29)			19.6 (15–30)		
Demographic and socioeconomic background variables						
Mother’s educational level, *n* (%)						
Low	581 (29.6)	2510 (29.6)	0.995	684 (30.9)	2583 (30.4)	0.651
Medium	871 (44.4)	3681 (42.9)	0.044	984 (44.4)	3681 (43.3)	0.342
High	441 (22.5)	1785 (21.1)	0.169	469 (21.2)	1766 (20.7)	0.679
Missing data	69 (3.52)	628 (7.41)	<0.001	80 (3.61)	479 (5.63)	<0.001
Father’s educational level, *n* (%)						
Low	685 (34.9)	2767 (32.7)	0.055	751 (33.9)	2865 (33.7)	0.856
Medium	746 (38.2)	3210 (37.9)	0.907	901 (40.6)	3214 (37.8)	0.014
High	390 (19.9)	1567 (18.5)	0.157	424 (19.1)	1609 (18.9)	0.818
Missing data	141 (7.19)	930 (10.97)	<0.001	141 (6.36)	821 (9.65)	<0.001
Mother’s earnings, EUR, mean (SD)^b^	17,542 (11,623)	17,725 (12,356)	0.558	17,601 (12,107)	17,484 (12,233)	0.694
Father’s earnings, EUR, mean (SD)^c^	24,869 (22,553)	23,989 (19,482)	0.090	23,227 (17,954)	23,698 (18,831)	0.306
Parent(s) born in a non-Nordic country, *n* (%)^d^	20 (1.04)	344 (4.24)	<0.001	32 (1.47)	360 (4.36)	<0.001
Outcome variables						
Employed (in November), *n* (%)^d^	1457 (75.8)	6519 (80.4)	<0.001	1818 (83.8)	7054 (85.4)	0.056
Earnings if employed (EUR), mean (SD)	22,466 (13,333)	23,175 (13,334)	0.066	32,202 (13,282)	34,129 (24,301)	0.001
Mediator variables						
Years of schooling, mean (SD)^e^	12.6 (2.08)	12.8 (2.12)	<0.001	12.2 (1.98)	12.3 (2.07)	0.001
Expected earnings in occupational field (EUR), mean (SD)^f^	3278 (782)	3351 (806)	<0.001	3529 (852)	3595 (891)	0.003
Having children, *n* (%)^d^	1052 (54.5)	4674 (57.6)	0.013	805 (37.1)	3297 (39.9)	0.016
Sickness benefits during the year, *n* (%)^b^	598 (31.0)	1575 (19.4)	<0.001	280 (12.9)	603 (7.3)	<0.001
If sickness benefits, number of episodes, median (min–max)^g^	1 (1–6)	1 (1–9)	0.056	1 (1–9)	1 (1–9)	0.155
If sickness benefits, number of days, median (min–max)^g^	63 (1–366)	37 (1–366)	<0.001	49 (1–366)	32 (1–366)	0.030
Inpatient care during the year, *n* (%)	475 (24.2)	1481 (17.5)	<0.001	262 (11.8)	283 (3.33)	<0.001
If inpatient care, number of episodes, median (min–max)	1 (1–13)	1 (1–16)	<0.001	1 (1–17)	1 (1–15)	0.003
If inpatient care, number of days, median (min–max)	5 (1–252)	3 (0.5–76)	<0.001	2 (0.5–200)	2 (0.5–365)	0.276
Cohort born in 1974–1979, n	*1021*	*4516*		*1164*	*4371*	
One or more outpatient care visits, *n* (%)	923 (90.4)	1650 (36.5)	<0.001	958 (82.3)	918 (21.0)	<0.001
Two or more outpatient care visits, *n* (%)	668 (65.4)	890 (19.7)	<0.001	509 (43.7)	404 (9.24)	<0.001
If outpatient care, number of visits, median (min–max)	3 (1–176)	2 (1–21)	<0.001	2 (1–216)	1 (1–15)	<0.001

^a^ *t* tests for means, Pearson χ^2^ for medians and test of proportions

^b^Data missing: 139 individuals with type 1 diabetes and 1027 individuals in the control group

^c^Data missing: 270 individuals with type 1 diabetes and 1606 individuals in the control group

^d^Data missing: 80 individuals with with type 1 diabetes and 616 individuals in the control group

^e^Data missing: 102 individuals with type 1 diabetes and 681 individuals in the control group

^f^Data missing: 386 individuals with type 1 diabetes and 1388 individuals in the control group

^g^Data missing: one individual with with type 1 diabetes and four individuals in the control group

Low education, compulsory schooling; medium education, upper secondary school education; high education, university education

As expected, the indicators for adult health showed that the diabetes group generally had more sick leave and received more in- and outpatient care compared with the control group. Among women with diabetes, the number receiving sickness benefits was higher (31% vs 19%; *p* < 0.001), and in this subgroup, the median duration of sickness benefits was longer compared with the control group (63 vs 37 days; *p* < 0.001). There was, however, no difference in the number of registered yearly episodes of sickness benefit. A similar tendency was seen among men. The proportion receiving inpatient care during the year was higher among women and men with diabetes compared to the control group (24% vs 18%, *p* < 0.001 and 12% vs 3%, *p* < 0.001, respectively), although 11% of women with diabetes vs 13% of women in the control group had a main diagnosis related to pregnancy, childbirth and puerperium. Among women receiving inpatient care, the median number of days was also higher compared with the control group (5 vs 3 days, *p* < 0.001). The largest difference was seen in the utilisation of outpatient care, where the proportion with at least two visits during the year was 65% vs 20%, *p* < 0.001, for women and 44% vs 9%, *p* < 0.001, for men.

**Fig. 3:**
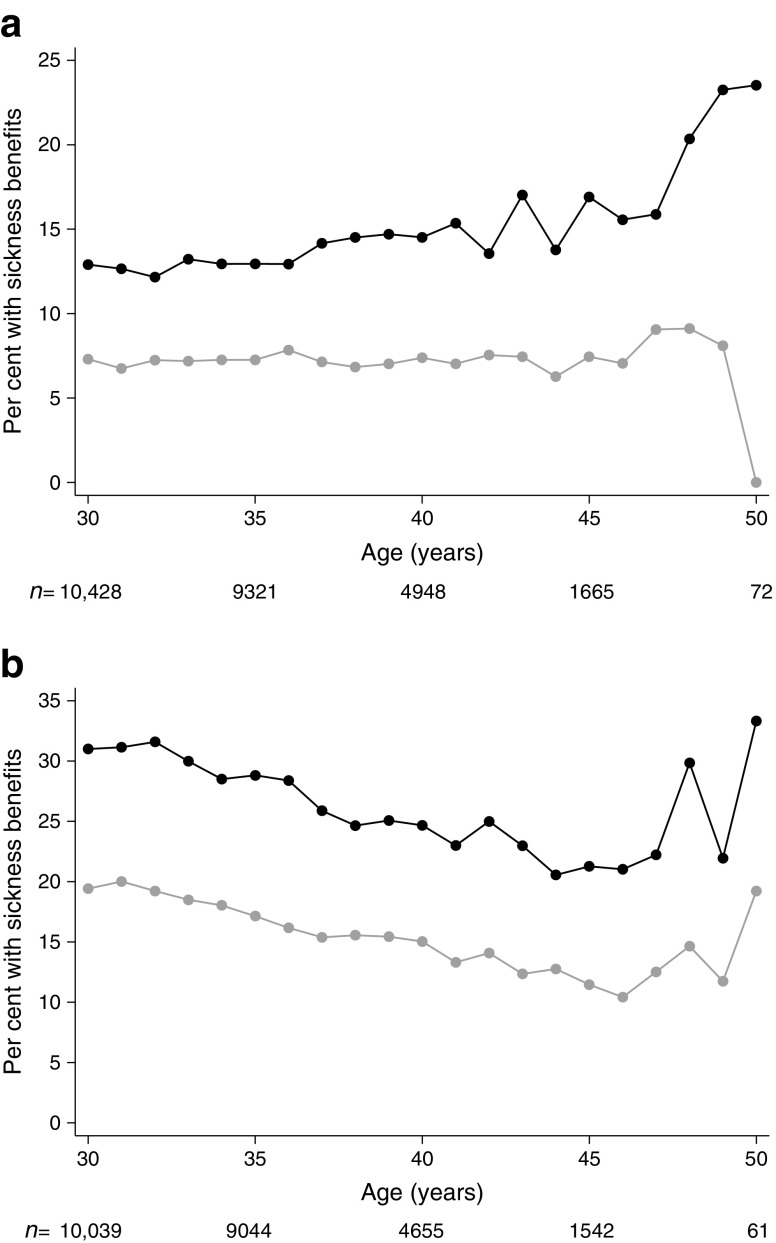
Proportion of men (**a**) and women (**b**) with sickness benefits between 30 and 50 years of age. Black, individuals with type 1 diabetes; grey, control group. The number of individuals at each age is shown below the graph

**Fig. 4:**
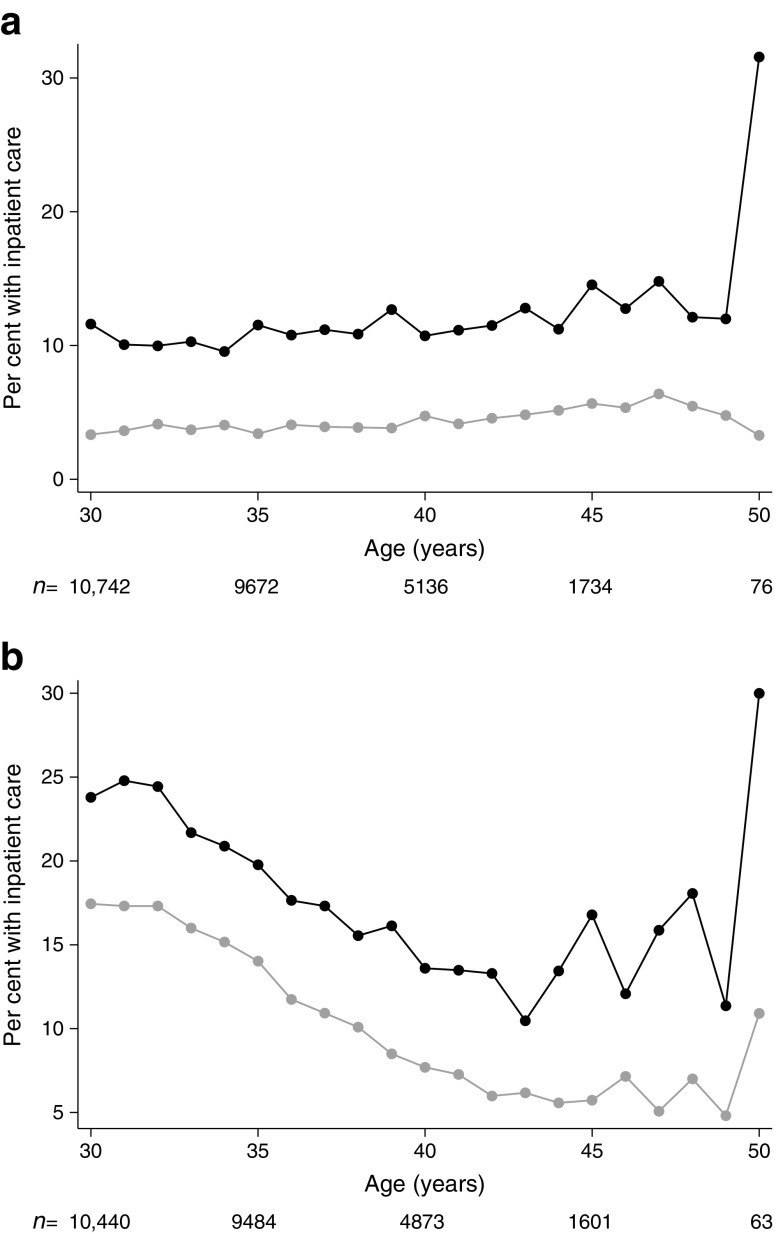
Proportion of men (**a**) and women (**b**) requiring inpatient care between 30 and 50 years of age. Black, individuals with type 1 diabetes; grey, control group. The number of individuals at each age is shown below the graph

**Fig. 5:**
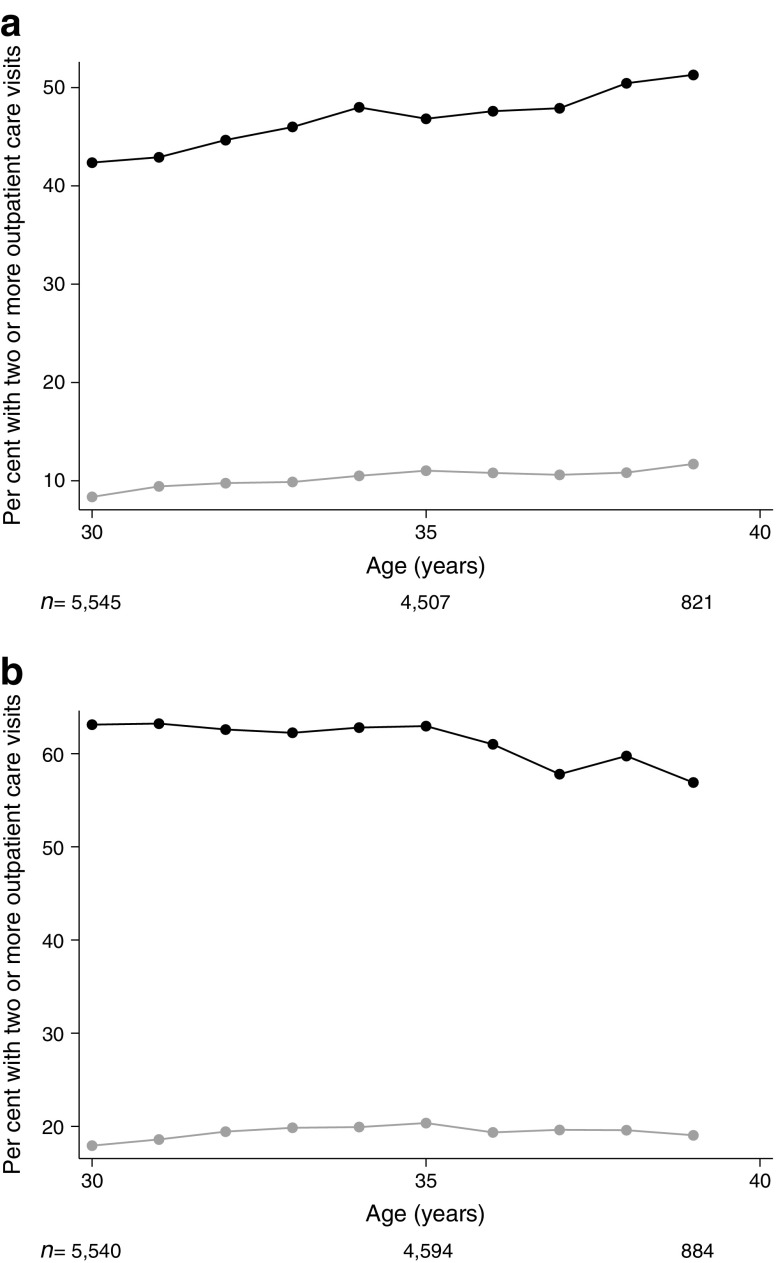
Proportion of men (**a**) and women (**b**) requiring two or more outpatient care visits per year between 30 and 39 years of age. Black, individuals with type 1 diabetes; grey, control group. The number of individuals at each age is shown below the graph

After 30 years of age, the difference between the type 1 diabetes and control groups was relatively stable in terms of the three health indicators (Figs 3, 4, 5), except for greater variability after 45 years of age due to fewer observations. Women, however, differed from men in that a considerably higher proportion of women received sickness benefits and inpatient care at a younger age, which was likely related to pregnancy and childbirth.

### The effect of type 1 diabetes on the four mediators

All of the mediators were affected by type 1 diabetes, both at 30 and 40 years of age, when controlling for confounders (Table 3). At 30 years of age, women and men with diabetes on average had −0.23 (<0.001) and −0.18 (<0.001) years less of schooling, respectively (Model 1), with a larger effect among those with longer disease duration, particularly among men (0.13 vs 0.25 after ≤24 and ≥25 years of duration (Model 2)). Furthermore, individuals with diabetes worked in occupations with a lower expected income on average (−2%, *p* < 0.001 and *p* = 0.001 at 30 years of age; −3%, *p* < 0.001 and *p* = 0.004 at 40 years of age for women and men, respectively) and were less likely to have children, which was particularly notable at 40 years of age (women OR 0.65 [95% CI 0.53, 0.79] and men OR 0.69 [95% CI 0.59, 0.80]).

**Table 3:** The effect of type 1 diabetes on potential mediators at 30 and 40 years of age

	Women			Men		
Mediator	Age 30 years		Age 40 years^a^	Age 30 years		Age 40 years^a^
	Model 1	Model 2	Model 1	Model 1	Model 2	Model 1
Years of schooling, *n* ^b^	9189	9189	4195	9568	9568	4464
Control (reference), β (*p*)						
Diabetes case	−0.23 (<0.001)		−0.27 (<0.001)	−0.18 (<0.001)		−0.21 (0.004)
Diabetes duration ≤24 years		−0.21 (0.002)			−0.13 (0.027)	
Diabetes duration ≥25 years		−0.26 (< 0.001)			−0.25 (< 0.001)	
Occupation, *n* ^b, c^	8629	8629	3971	9131	9131	4275
Control (reference), β (*p*)						
Diabetes case	−0.02 (<0.001)		−0.03 (<0.001)	−0.02 (0.001)		−0.03 (0.004)
Diabetes duration ≤24 years		−0.02 (0.019)			−0.01 (0.048)	
Diabetes duration ≥25 years		−0.03 (<0.001)			−0.02 (0.001)	
Family formation (having children), *n* ^d^	9220	9220	4200	9609	9609	4469
Control (reference), OR (95% CI)						
Diabetes case	0.88 (0.79, 0.98)		0.65 (0.53, 0.79)	0.88 (0.80, 0.98)		0.69 (0.59, 0.80)
Diabetes duration ≤24 years		0.88 (0.76, 1.01)			0.86 (0.75, 0.98)	
Diabetes duration ≥25 years		0.88 (0.76, 1.01)			0.92 (0.80, 1.07)	
Sickness benefits, *n* ^d^	9220	9220	4200	9609	9609	4469
Control (reference), OR (95% CI)						
Diabetes case	1.88 (1.67, 2.11)		1.90 (1.58, 2.28)	1.86 (1.59, 2.19)		2.10 (1.66, 2.64)
Diabetes duration ≤24 years		1.92 (1.65, 2.24)			1.66 (1.36, 2.03)	
Diabetes duration ≥25 years		1.84 (1.57, 2.15)			2.16 (1.74, 2.68)	
Inpatient care, *n* ^d^	9215	9215	4195	9603	9603	4463
Control (reference), OR (95% CI)						
Diabetes case	1.43 (1.27, 1.62)		1.70 (1.34, 2.15)	4.04 (3.36, 4.87)		2.57 (1.99, 3.32)
Diabetes duration ≤24 years		1.47 (1.25, 1.72)			3.82 (3.05, 4.79)	
Diabetes duration ≥25 years		1.39 (1.18, 1.65)			4.35 (3.41, 5.56)	
Sample born in 1974–1979						
Two or more outpatient care visits, *n* ^d^	4941	4941		5030	5030	
Control (reference), OR (95% CI)						
Diabetes case	7.74 (6.62, 9.05)		–	8.77 (7.62, 10.08)		–
Diabetes duration ≤24 years		7.22 (5.76, 9.08)			5.99 (4.83, 7.41)	
Diabetes duration ≥25 years		8.09 (6.68, 9.82)			10.02 (8.25, 12.2)	

^a^The duration of type 1 diabetes among individuals at 40 years of age ranged from 25 to 36 years

^b^OLS regression

^c^log_*e*_ (Expected earnings) in occupational field

^d^Logistic regression

Data adjusted for parents’ education and income; having a parent born in a non-Nordic country; and calendar year

Living with type 1 diabetes increased the likelihood of receiving sickness benefits (women OR 1.88 [95% CI 1.67, 2.11] and men 1.86 [95% CI 1.59, 2.19]) at 30 years of age (Model 1). For men, the effect was slightly larger among those with a longer disease duration of type 1 diabetes. Similarly, living with type 1 diabetes increased the use of in- and outpatient care, particularly for having two or more outpatient care visits per year (women OR 7.74 [95% CI 6.62, 9.05] and men 8.77 [95% CI 7.62, 10.08]). Generally, the duration of living with type 1 diabetes had little impact on the magnitude of the effect of diabetes for women while it increased the effect on most of the mediators for men (Model 2).

### Breaking down the effect of type 1 diabetes on employment and earnings

Using the Sobel–Goodman mediation test, we established that all four mediators investigated in the present study were in fact significant mediators between diabetes and employment and earnings when assessed separately. Table 4 presents the main results of the mediation analysis; Model 1 for the total sample between 30 and 50 years of age including sickness benefits and inpatient care as health indicators and Model 2 for the sub-sample born in 1974–1979 between 30 and 39 years of age also including outpatient care (two visits or more per year). In women, diabetes had a negative effect on both employment (OR 0.68 [95% CI 0.62, 0.76]) and earnings among those employed (−6%, *p* < 0.001) during the 20 year period (Model 1, Table 4). In the analysis of employment, the share of the total effect of diabetes being mediated was 34%, with occupational field accounting for the largest part of the effect (16%, Model 1). The inclusion of outpatient care in the analysis further increased the share of the effect mediated to 75% (Model 2). In the analysis of earnings for those employed, the health indicators appeared to play an essential role and including them together with the other mediators accounted for the total effect of diabetes on earnings, even totalling more than 100%. However, the mediation effect of family formation on earnings was negative, which implies that part of the total effect of diabetes is reduced by its effect on family formation.

**Table 4:** Direct and indirect effects of type 1 diabetes on employment and earnings at 30–50 years of age in individuals born in 1962–1979 and at 30–39 years of age for individuals born in 1974–1979

	Women				Men			
Effect	Model 1 Sample born in 1962–1979		Model 2 Sample born in 1974–1979		Model 1 Sample born in 1962–1979		Model 2 Sample born in 1974–1979	
	Employment OR (95% CI)	log_*e*_ (Earnings) if employed β (*p*)	Employment OR (95% CI)	log_*e*_ (Earnings) if employed β (*p*)	Employment OR (95% CI)	log_*e*_ (Earnings) if employed β (*p*)	Employment OR (95% CI)	log_*e*_ (Earnings) if employed β (*p*)
Individuals (observations)	8923 (92,331)	8714 (80,859)	4790 (34,822)	4629 (30,671)	9319 (97,913)	8183 (89,638)	4914 (35,807)	4799 (32,736)
Total effect	0.68 (0.62, 0.76)	−0.06 (<0.001)	0.69 (0.59, 0.81)	−0.03 (0.078)	0.76 (0.67, 0.86)	−0.08 (<0.001)	0.71 (0.59, 0.84)	−0.08 (< 0.001)
Direct effect	0.78 (0.70, 0.87)	0.01 (0.239)	0.91 (0.77, 1.08)	0.02 (0.371)	0.91 (0.80, 1.03)	−0.03 (<0.001)	0.92 (0.77, 1.11)	−0.03 (0.017)
Indirect effect	0.88 (0.87, 0.90)	−0.07 (<0.001)	0.76 (0.70, 0.82)	−0.05 (<0.001)	0.83 (0.82, 0.85)	−0.05 (<0.001)	0.77 (0.71, 0.83)	−0.05 (< 0.001)
Share (%) of total effect due to mediators	33.71	122.60	74.75	153.70	65.14	61.32	76.58	59.74
Share (%) of total effect mediated via:								
Education	9.92	3.30	11.67	5.47	4.97	4.26	6.16	3.81
Occupation	15.50	36.80	23.06	75.96	19.68	19.58	19.59	20.43
Family formation	4.40	−18.01	5.45	−49.35	15.59	3.11	8.18	0.39
Adult health								
Sickness benefits during the year	−2.02	72.99	−12.37	110.34	5.61	25.88	− 0.67	17.83
Inpatient care during the year	5.91	27.52	2.89	32.45	19.30	8.49	17.19	7.30
Two or more outpatient visits during the year			44.01	−21.15			26.13	9.98

Logistic and OLS regression with clustered and bootstrapped standard errors and 95**%** CIs

Adjusted for parents’ education and income; having a parent born in a non-Nordic country; and calendar year

Log_*e*_ (Earnings), logarithm of annual labour earnings

In men, the effect of diabetes on employment was OR 0.76 (95% CI 0.67, 0.86) and −8% (*p* < 0.001) on earnings if employed at 30–50 years of age (Model 1, Table 4). The share of the effect on employment, explained by the mediators, was 65% (Model 1) and 77% (Model 2) and the direct effect of diabetes was no longer significant in either of the models. For earnings, the mediators accounted for approximately 60% of the total diabetes effect but a −3% (*p* = 0.017) effect remained after including outpatient care as an additional mediator (Model 2). Similar to women, occupational field and the health indicators accounted for the largest part of the effect of diabetes in men. However, unlike for women, no reverse mediation effect of family formation was observed on earnings. Instead, 0–3% of the effect was mediated through family formation depending on model specification.

ESM Results (ESM Tables 2–5) present the mediation effect of each mediator when examined individually together with additional contributions of each mediator when added one by one.

### The effect of socioeconomic background

The results presented in Table 5 indicate that the effect of type 1 diabetes was relatively stable across socioeconomic background. None of the interactions between diabetes and having parents with a low educational level or low income was significant for either employment or earnings, a result that remained when testing alternative definitions of family educational level and income.

**Table 5:** Effect of type 1 diabetes on employment and earnings at 30–50 years of age, and interactions with having parents with a low educational level and low income

	Women		Men	
	Employment OR (95% CI)	log_*e*_ (Earnings) if employed β (*p*)	Employment OR (95% CI)	log_*e*_ (Earnings) if employed β (*p*)
Individuals, *n* (observations)	9292 (97,177)	8783 (82,147)	9678 (101,543)	9262 (89,867)
Diabetes	0.71 (0.62, 0.82)	−0.05 (0.012)	0.68 (0.59, 0.80)	−0.07 (<0.001)
Low parental education	0.88 (0.77, 1.01)	−0.06 (<0.001)	0.99 (0.85, 1.16)	−0.07 (<0.001)
Diabetes × low parental education	0.98 (0.74, 1.30)	0.04 (0.278)	0.99 (0.72, 1.36)	−0.05 (0.126)
Low parental income	0.79 (0.70, 0.88)	0.00 (0.795)	0.70 (0.62, 0.80)	−0.06 (<0.001)
Diabetes × low parental income	0.81 (0.64, 1.02)	−0.05 (0.135)	1.11 (0.86, 1.43)	0.01 (0.776)

Data adjusted for having a parent born in a non-Nordic country, and calendar year

log_*e*_ (Earnings), logarithm of annual labour earnings

## Discussion

This study sheds light on the complex mechanisms between onset of type 1 diabetes during childhood and future labour market outcomes. Results show that type 1 diabetes negatively effects both employment and earnings at 30–50 years of age and a major part of this effect is mediated by health, occupation, education and family formation. The three health measures related to absenteeism and in- and outpatient care accounted for more than half of the indirect effect of type 1 diabetes on earnings, indicating that a large part of the effect is driven by increased absenteeism and reduced work capacity, which may be associated with diabetes-related complications. Part of the absenteeism may be due to longer periods of sick leave in the case of non-diabetes-related illnesses such as infections and surgery that affect the metabolic control.

Occupation also accounted for a large part of the effect of type 1 diabetes on employment and earnings. This finding may be related to personal choice, as individuals with type 1 diabetes may select particular jobs as a consequence of decreased flexibility due to the daily self-management of type 1 diabetes, including blood glucose monitoring, insulin injection and strict routines for diet and exercise. It could also be related to constraints in career opportunities, as the risk of hypoglycaemia may restrict access to some types of jobs because of safety issues.

Education mediated 10–12% of the effect on employment among women, depending on model specification, but accounted for a smaller part of the effect on earnings (3–6%) for both women and men and on employment among men (6%). This is in line with previous studies reporting that the effect of childhood health remains relatively stable when controlling for education [37, 38].

In line with previous findings [9, 10], our data showed that individuals with type 1 diabetes were less likely to have children. Family formation reduced part of the diabetes effect for women, potentially because not having children increases time available for working and career opportunities. This tendency was not observed in men, for whom family formation accounted for a small part of the effect of diabetes on employment and earnings, perhaps because men are not as physically affected by pregnancy and childbirth to the same extent as women. The small mediation effect found for men could instead be due to a link between diabetes-related complications and decreased fertility [39].

For men, part of the effect of diabetes on employment and earnings (23% and 40%, respectively) could not be attributed to any of the mediators included in this study, suggesting that there may be other characteristics of the disease that hamper productivity and career opportunities that were not possible to extract from our data. Living with type 1 diabetes involves a number of less obvious burdens of self-care not requiring in- or outpatient specialist care or resulting in sick leave longer than 14 days, such as episodes of hypoglycaemia or depression [40]. Additionally, potential discrimination against people with diabetes [41, 42] may not have been picked up by any of the mediators selected for our investigation.

For women, the interpretation of the results is complex as the estimated mediation effect on earnings totalled more than 100%. This can occur when other mediators exist that impact in the opposite direction to the investigated relationship [32, 33], such as family formation in this instance, since positive and negative mediators can offset each other. In such situations, results should be interpreted with caution [33]. For employment, however, 25% of the effect of diabetes among women was not explained by the studied mediators.

A common hypothesis is that the effect of poor health may be larger in lower socioeconomic groups as individuals within these groups may have lower ability to compensate for poor health outcomes [43]. We found no evidence of this in our study. This may indicate either that the effect of diabetes is related to factors that cannot be compensated for by parental higher education and income level, or it may reflect that the systems of healthcare and education in Sweden seemingly compensate for parental socioeconomic status.

A major strength of this study is the use of longitudinal, individual-level data from national population registers, allowing us to follow the study population through a large part of their working life (across 30–50 years of age). Additionally, the study is based on over 4000 individuals with type 1 diabetes and over 17,000 matched individuals within our control group. This rich dataset allowed us to explore the role of several potential mediators and to adjust for potential confounding due to socioeconomic background factors. Furthermore, the use of a formal mediation analysis enabled us to break down the total effect of diabetes to investigate the contribution of each mediator when assessed jointly. This type of analysis has not been performed before in the case of labour market consequences of type 1 diabetes. Previous studies have analysed the underlying mechanisms by including them in the regression of labour market outcomes to explore how this alters the estimated effect of diabetes [2, 5].

Some limitations to this study should be noted. As data on occupational field were available from 2001, the information regarding area of work for individuals with missing information at an early age was assumed to be similar later in life. Moreover, follow-up data for the full sample were available only for the 30–34 years age bracket, given that the youngest participants were born in 1979. The sample size was thereafter reduced for each year of follow-up, which should be kept in mind when interpreting the results. Compared with other studies in this area, the sample size can, however, be considered large even in the older age groups (for example, we had more than 1800 individuals with type 1 diabetes followed at least up to 40 years of age). Another potential limitation, which can never be ruled out in observational studies, is that there may exist confounding factors impacting both the onset of type 1 diabetes, and the mediators and outcomes. The current consensus is that type 1 diabetes is triggered by a complex chain of genetic and environmental events [44–47] that the individual is unable to influence or anticipate beforehand. Previous studies also show that bias in analyses of education and labour market outcomes due to confounding from genetic or perinatal factors, as well as socioeconomic and demographic factors, is likely to be small, if present at all [2, 6]. There may, however, still exist confounders between the mediators and the outcomes that could bias the result of the mediation analysis [34]. To account for this, we controlled for demographic and socioeconomic background characteristics and assessed the robustness of each of the mediators using sensitivity analysis.

The results of this study represent a setting where healthcare is mainly tax-financed and where healthcare for children and all insulin is free of charge [48]. Long-term consequences of type 1 diabetes may be different in settings where the financial burden of healthcare is borne by people with this disease to a larger extent.

Understanding the mechanisms between type 1 diabetes and labour market outcomes is crucial for tailoring interventions to reduce the long-term consequences of the disease. Our results show the importance of maintaining good health in adulthood; but also that there are other important factors that need to be considered for reducing labour market effects of type 1 diabetes, particularly those related to choice of occupation.

## Electronic supplementary material

ESM (PDF 571 kb)

## Abbreviations

KHB method: Karlson, Holme and Breen method
LISA: Longitudinal Integration Database for Health Insurance and Labour Market Studies
OLS: Ordinary least square
SCDR: Swedish Childhood Diabetes Register
