# Supplementary material for: Why childhood-onset type 1 diabetes impacts labour market outcomes: a mediation analysis
Source: Diabetologia. 2017 Nov 23;61(2):342–53. doi: 10.1007/s00125-017-4472-3 (PMC6448960; doi:10.1007/s00125-017-4472-3)
Supplement: Supplementary file 1 — (PDF 571 kb) [file 125_2017_4472_MOESM1_ESM.pdf]

Electronic supplementary material (ESM) methods

Sensitivity analysis

In situations when both the mediator (M) and the outcome (Y) variables are continuous and linear regression is used, the mediation effect can be estimated as follows:

$$M_i=\alpha_1+\beta_1 T_i+\xi^{T}{}_1 X_i+\varepsilon_{i1} \quad (1)$$

$$Y_i=\alpha_2+\beta_2 T_i+\gamma M_i+\xi^{T}{}_2 X_i+\varepsilon_{i2} \quad (2)$$

taking the product of the coefficients on the treatment variable in Model 1 with the coefficient on the mediator model in Model 2, $\beta_2\gamma$.

The estimated mediation effect can be considered causal under two main assumptions, defined by the sequential ignorability (SI) assumption:

1. The treatment variable is conditionally independent of unobservables, given background covariates.
2. The mediator variable is conditionally independent of unobservables, given background covariates and the treatment variable.

As these assumptions may be considered too strong for the typical situations in which causal mediation is studied, sensitivity analysis has been proposed. For example, in randomized controlled trials the first assumption holds, as the treatment assignment is random, but this does not mean that assumption 2 holds, as the mediators may not be randomly assigned. In such cases, there may exist pre- and post-treatment confounders between the mediator and the outcome. It is not possible to test this assumption, but through sensibility analysis we can investigate how robust the results are to the violation of the SI assumption [34, 35].

Using the *medsens* command included in the Mediation package in Stata (StataCorp, College Station, TX, USA) it is possible to assess the sensitivity of an estimated mediation effect of single mediators to unmeasured confounding [36]. This sensitivity analysis is based on the correlation across the two error terms in models 1 and 2 (ε_{i1} and ε_{i1}). Such correlation can exist if there are omitted variables that affect both the mediator and the outcome because these variables will then be part of both error terms. If the SI assumption holds, then the correlation of the error term (ρ) will be zero and non-zero values mean that the SI assumption is not fully satisfied. The sensitivity analysis addresses the question of how large the ρ will have to be for the mediation effect to disappear. The results may be considered sensitive to violation of the SI assumption if a small departure from zero in ρ produces a substantially different mediation effect [34, 35]. There is no absolute threshold for

the magnitude of ρ, but it can be interpreted in relation to the corresponding ρ from other studies [35].

There is to date no available sensitivity analysis method that can be applied for situations with multiple mediators or in a panel data setting [32, 35]. To still be able to provide some indication of the sensitivity of our results due to potential validation of the SI assumption, we present the estimated ρ for the mediation analysis of each of the mediators separately at the age of 30 years (ESM Table 1). As recommended in Imai, Keele and Tingley [35], we used 1,000 simulations for each step.

For women, the ρ for the different mediators ranged from -0.37 to 0.23, compared with -0.17 to 0.25 for men. The results show that for education among women, for example, ρ would have to equal 0.1379 for the true mediated effect to be zero. ESM Figures 1 and 2 graphically illustrate the sensitivity analysis by plotting the estimated average mediation effect and 95% confidence intervals (CIs) as a function of ρ. The dashed line shows the average mediation effect at different values of ρ.

ESM Table 1: Sensitivity parameter (ρ)

Mediator	Women	Men
Years of schooling	0.1379	0.1498
Occupation	0.2250	0.2454
Family formation	-0.3683	-0.1385
Sickness benefits	-0.2724	-0.1729
Inpatient care	-0.2611	-0.0617

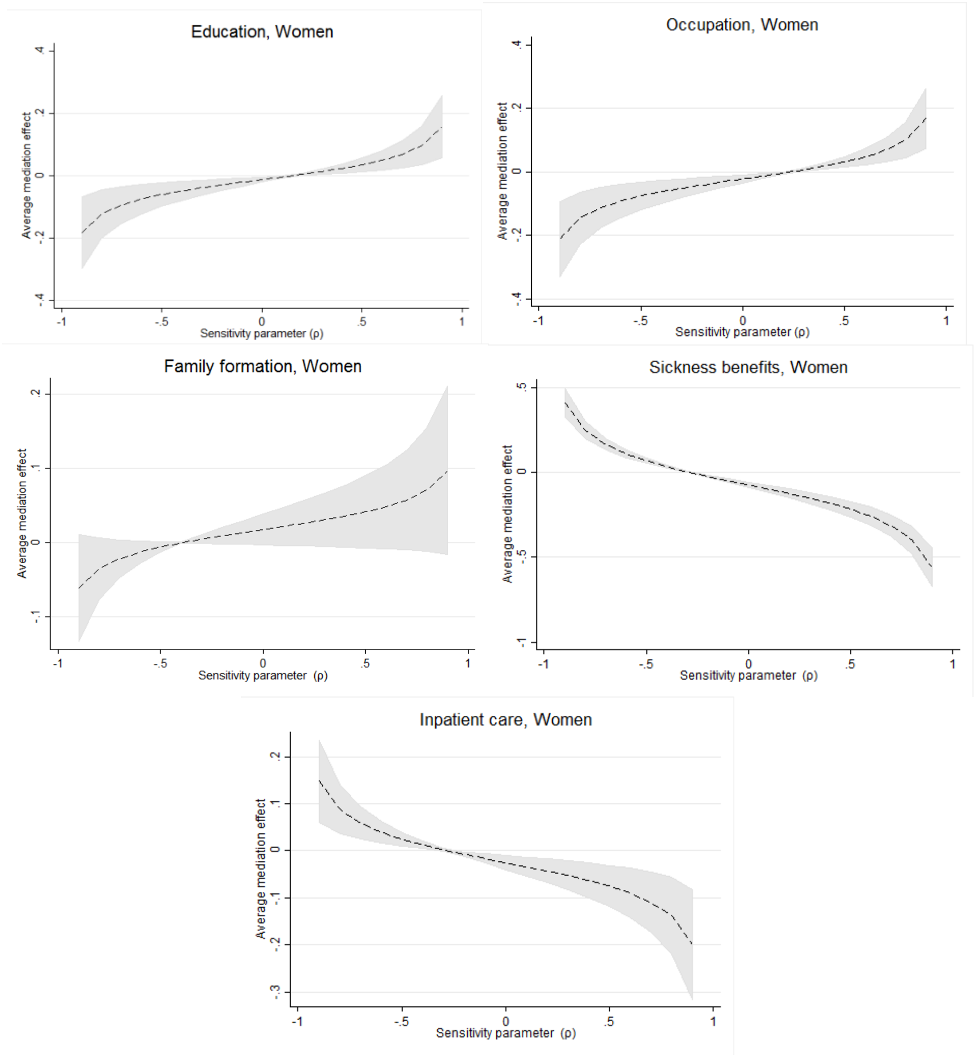

ESM Figure 1: Graphically illustrating the sensitivity analysis for women by plotting the estimated average mediation effect and 95% confidence intervals (CIs) as a function of ρ.

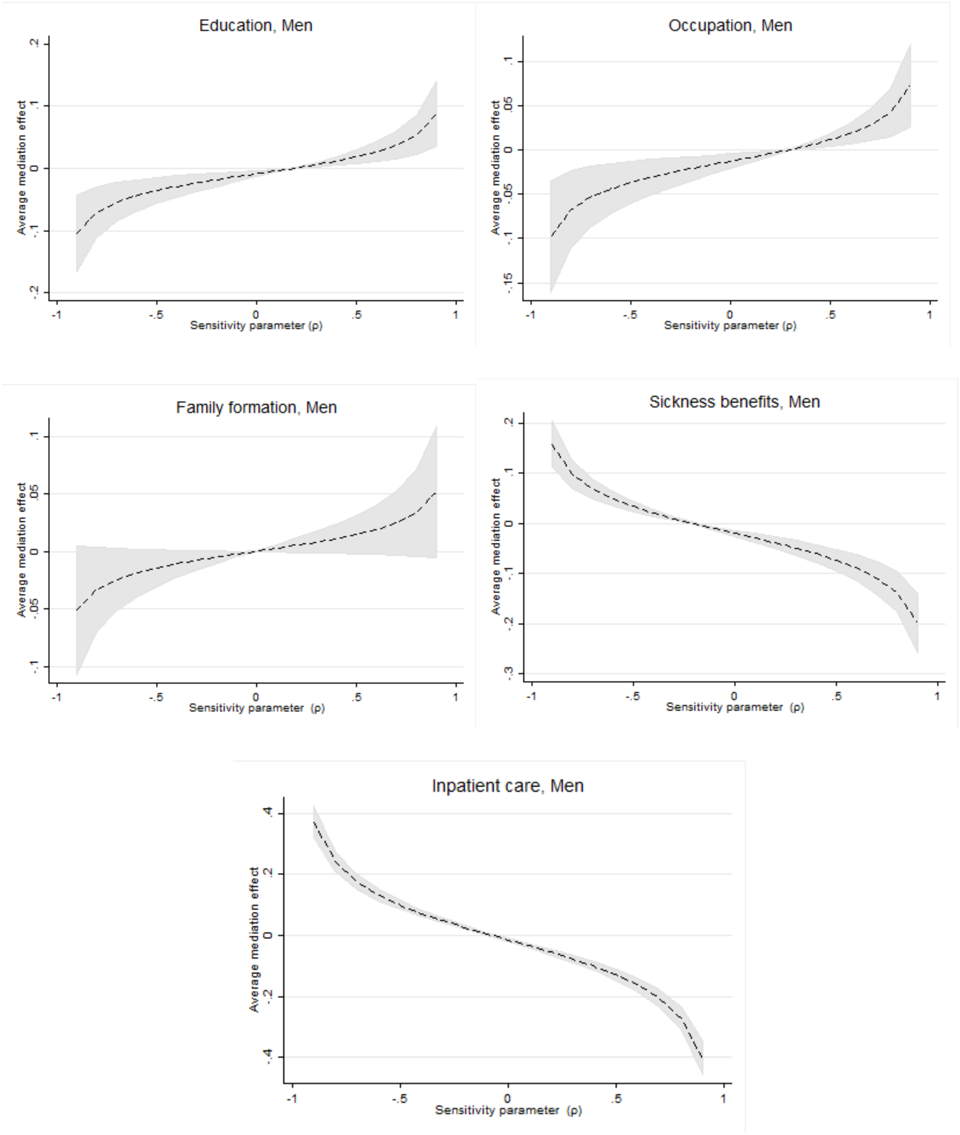

ESM Figure 2: Graph illustrating the sensitivity analysis for men by plotting the estimated average mediation effect and 95% confidence intervals (CIs) as a function of ρ.

Electronic supplementary material (ESM) results

ESM Table 2: Direct and indirect effects of type 1 diabetes on employment and earnings among women 30–50 years old (logistic and ordinal least square (OLS) regression with clustered and bootstrapped standard errors and 95% confidence intervals (CIs))

	Employment OR (95% CI) Individuals = 8,923; observations = 92,331						Earnings if employed β (p) Individuals = 8,714; observations = 80,859					
	Education	Occupa-tion	Family formation	Sickness benefits	Inpatient care	Outpatient care (≥2 visits)[a]	Education	Occupation	Family formation	Sickness benefits	Inpatient care	Outpatient care (≥2 visits)[b]
Total effect	0.69 (0.62, 0.77)	0.68 (0.61, 0.77)	0.70 (0.62, 0.78)	0.70 (0.63, 0.78)	0.70 (0.63, 0.78)	0.71 (0.60, 0.84)	-0.06 (<0.001)	-0.06 (<0.001)	-0.06 (<0.001)	-0.06 (<0.001)	-0.06 (<0.001)	-0.03 (0.129)
Direct effect	0.73 (0.65, 0.81)	0.73 (0.66, 0.82)	0.71 (0.63, 0.79)	0.71 (0.64, 0.80)	0.71 (0.64, 0.79)	0.83 (0.70, 0.99)	-0.05 (0.001)	-0.04 (0.010)	-0.07 (<0.001)	0.01 (0.622)	-0.04 (0.014)	0.04 (0.054)
Indirect effect	0.95 (0.93, 0.97)	0.93 (0.90, 0.96)	0.99 (0.98, 1.00)	0.98 (0.97, 0.99)	0.98 (0.98, 0.99)	0.85 (0.82, 0.89)	-0.01 (0.002)	-0.02 (<0.001)	0.01 (0.001)	-0.07 (<0.001)	-0.02 (<0.001)	-0.08 (<0.001)
Share (%) of total effect due to mediators	14.25	19.91	3.04	5.28	4.78	45.84	17.32	40.91	-24.23	111.59	39.57	231.64

[a]Sample born in 1974–1979 and studied at the ages of 30–39: individuals with type 1 diabetes = 4,790; observations = 34,822. [b]Sample born in 1974–1979 and studied at the ages of 30–39: individuals = 4,629; observations = 30,671. Adjusted for parental education and income; having a parent born in a non-Nordic country; and calendar year. OR = odds ratio.

ESM Table 3: Direct and indirect effects of type 1 diabetes on employment and earnings among men aged 30–50 years (logistic and ordinal least square (OLS) regression with clustered and bootstrapped standard errors and 95% confidence intervals (CIs))

	Employment OR (95% CI) Individuals = 9,319; observations = 97,913						Earnings if employed β (p) Individuals = 8,183; observations = 89,638					
	Education	Occupa-tion	Family formation	Sickness benefits	Inpatient care	Outpatient care (≥2 visits)[a]	Education	Occupation	Family formation	Sickness benefits	Inpatient care	Outpatient care (≥2 visits)[b]
Total effect	0.75 (0.66, 0.84)	0.73 (0.64, 0.83)	0.75 (0.66, 0.85)	0.76 (0.67, 0.86)	0.77 (0.68, 0.87)	0.71 (0.59, 0.85)	-0.08 (<0.001)	-0.08 (<0.001)	-0.08 (<0.001)	-0.08 (<0.001)	-0.08 (<0.001)	-0.08 (<0.001)
Direct effect	0.77 (0.68, 0.87)	0.78 (0.69, 0.88)	0.78 (0.69, 0.88)	0.79 (0.69, 0.89)	0.82 (0.73, 0.93)	0.86 (0.71, 1.03)	-0.07 (<0.001)	-0.06 (<0.001)	-0.07 (<0.001)	-0.05 (<0.001)	-0.06 (0.001)	-0.04 (0.016)
Indirect effect	0.97 (0.96, 0.99)	0.93 (0.90, 0.97)	0.96 (0.94, 0.98)	0.96 (0.95, 0.97)	0.93 (0.92, 0.94)	0.83 (0.78, 0.87)	-0.01 (0.004)	-0.02 (0.001)	-0.00 (0.002)	-0.03 (<0.001)	-0.02 (<0.001)	-0.04 (<0.001)
Share (%) of total effect due to mediators	9.74	21.40	15.40	14.41	26.13	55.99	11.17	22.36	4.13	33.45	21.58	52.50

[a]Sample born in 1974–1979 and studied at the ages of 30–39: individuals = 4,914; observations = 35,807. [b]Sample born in 1974–1979 and studied at the ages of 30–39: individuals = 4,799; observations = 32,736. Adjusted for parental education and income; having a parent born in a non-Nordic country; and calendar year. OR = odds ratio.

ESM Table 4: Direct and indirect effects of type 1 diabetes on employment and earnings among women aged 30–50 (logistic and ordinal least square (OLS) regression with clustered and bootstrapped standard errors and 95% confidence intervals (CIs))

	Employment OR (95% CI) Individuals = 8,923; observations = 92,331						Earnings if employed β (p) Individuals = 8,714; observations = 80,859					
	Model 1	Model 2	Model 3	Model 4	Model 5	Model 6[a]	Model 1	Model 2	Model 3	Model 4	Model 5	Model 6[b]
Total effect	0.69 (0.62, 0.77)	0.68 (0.61, 0.76)	0.68 (0.61, 0.76)	0.68 (0.61, 0.76)	0.68 (0.61, 0.76)	0.69 (0.59, 0.81)	-0.06 (<0.001)	-0.06 (<0.001)	-0.06 (<0.001)	-0.06 (<0.001)	-0.06 (<0.001)	-0.03 (0.078)
Direct effect	0.73 (0.65, 0.81)	0.75 (0.67, 0.83)	0.76 (0.68, 0.85)	0.77 (0.68, 0.86)	0.78 (0.70, 0.87)	0.91 (0.77, 1.08)	-0.05 (0.001)	-0.03 (0.014)	-0.05 (<0.001)	0.01 (0.425)	0.01 (0.271)	0.02 (0.371)
Indirect effect	0.95 (0.93, 0.97)	0.91 (0.88, 0.94)	0.90 (0.86, 0.93)	0.89 (0.86, 0.92)	0.88 (0.87, 0.90)	0.76 (0.70, 0.82)	-0.01 (0.002)	-0.03 (<0.001)	0.011 (0.118)	-0.07 (<0.001)	-0.07 (<0.001)	-0.05 (<0.001)
Share (%) of total effect due to mediators	14.25	24.22	28.71	30.55	33.71	74.75	17.32	43.89	19.14	116.80	122.60	153.70
Share (%) of total effect mediated via:												
▪ Education	14.25	9.21	9.61	9.57	9.92	11.67	17.32	5.01	3.36	1.85	3.30	5.47
▪ Occupation		15.01	15.16	15.12	15.50	23.06		38.88	38.20	36.06	36.80	75.96
▪ Family formation			3.94	3.99	4.40	5.45			-22.31	-20.57	-18.01	-49.35
▪ Adult health												
- Sickness benefits during the year				1.87	-2.02	-12.37				99.46	72.99	110.34
- Inpatient care during the year					5.91	2.89					27.52	32.45
- Two or more outpatient visits during the year						44.01						-21.15

[a]Sample born in 1974–1979 and studied at the ages of 30–39: individuals = 4,790; observations = 34,822. [b]Sample born in 1974–1979 and studied at the ages of 30–39: individuals = 4,629; observations = 30,671. Adjusted for parental education and income; having a parent born in a non-Nordic country; and calendar year. OR = odds ratio.

ESM Table 5: Direct and indirect effects of type 1 diabetes on employment and earnings among men at the ages of 30–50 (logistic and ordinal least square (OLS) regression with clustered and bootstrapped standard errors and 95% confidence intervals (CIs))

	Employment OR (95% CI) Individuals = 9,319; observations = 97,913						Earnings if employed β (p) Individuals = 8,183; observations = 89,638					
	Model 1	Model 2	Model 3	Model 4	Model 5	Model 6[a]	Model 1	Model 2	Model 3	Model 4	Model 5	Model 6[b]
Total effect	0.75 (0.66, 0.84)	0.73 (0.64, 0.83)	0.73 (0.64, 0.83)	0.74 (0.65, 0.84)	0.76 (0.67, 0.86)	0.71 (0.59, 0.84)	-0.08 (<0.001)	-0.08 (<0.001)	-0.08 (<0.001)	-0.08 (<0.001)	-0.08 (<0.001)	-0.08 (<0.001)
Direct effect	0.77 (0.68, 0.87)	0.79 (0.69, 0.89)	0.82 (0.72, 0.93)	0.85 (0.75, 0.96)	0.91 (0.80, 1.03)	0.92 (0.77, 1.11)	-0.07 (<0.001)	-0.06 (<0.001)	-0.06 (<0.001)	-0.04 (<0.001)	-0.03 (<0.001)	-0.03 (0.017)
Indirect effect	0.97 (0.96, 0.99)	0.93 (0.90, 0.96)	0.89 (0.85, 0.93)	0.87 (0.83, 0.91)	0.83 (0.82, 0.85)	0.77 (0.71, 0.83)	-0.01 (0.004)	-0.02 (0.001)	-0.02 (<0.001)	-0.04 (<0.001)	-0.05 (<0.001)	-0.05 (<0.001)
Share (%) of total effect due to mediators	9.74	23.21	36.78	47.25	65.14	76.58	11.17	25.30	28.46	54.93	61.32	59.74
Share (%) of total effect mediated via:												
▪ Education	9.74	3.65	4.98	4.81	4.97	6.16	11.17	4.71	5.00	4.31	4.26	3.81
▪ Occupation		19.57	17.94	18.19	19.68	19.59		20.59	20.28	19.58	19.58	20.43
▪ Family formation			13.87	14.41	15.59	8.18			3.18	3.14	3.11	0.39
▪ Adult health												
- Sickness benefits during the year				9.85	5.61	-0.67				27.91	25.88	17.83
- Inpatient care during the year					19.30	17.19					8.49	7.30
- Two or more outpatient visits during the year						26.13						9.98

[a]Sample born in 1974–1979 and studied at the ages of 30–39: individuals = 4,914; observations = 35,807. [b]Sample born in 1974–1979 and studied at the ages of 30–39: individuals = 4,799; observations = 32,736. Adjusted for parental education and income; having a parent born in a non-Nordic country; and calendar year. OR = odds ratio.
